# Prevalence of High-Risk Human Papillomavirus (HR-HPV) Genotypes and Multiple Infections in Cervical Abnormalities from Northern Xinjiang, China

**DOI:** 10.1371/journal.pone.0160698

**Published:** 2016-08-05

**Authors:** Lina Wang, Pengyan Wang, Yan Ren, Jingyun Du, Jianjun Jiang, Xuesong Jia, Chuangfu Chen, Yuanzhi Wang

**Affiliations:** 1 School of Animal Science and Technology, Shihezi University, Shihezi, Xinjiang Uygur Autonomous Region, People’s Republic of China; 2 School of Medicine, Shihezi University, Shihezi, Xinjiang Uygur Autonomous Region, People’s Republic of China; 3 First Affiliated Hospital of the School of Medicine, Shihezi University, Shihezi, Xinjiang Uygur Autonomous Region, People’s Republic of China; 4 Maternal and Child Health Hospital, Baoding, Hebei Province, People’s Republic of China; Istituto Nazionale Tumori, ITALY

## Abstract

Multiple human papillomavirus (HPV) genotypes often coexist within the cervical epithelia and are frequently detected together in various grades of the cervical neoplasia. To date, only a few reports exist on multiple HPV infections of HPV in Xinjiang Uygur Autonomous Region (XUAR). In the present study, we investigated the prevalence of High-Risk HPV (HR-HPV) genotypes and multiple infections. Cervical cytology samples were collected from 428 women who presented cervical abnormalities. Genotyping of HPV was performed by polymerase chain reaction–sequencing based typing (PCR-SBT) using consensus primers and specific primers. Of them, 166 samples were positive for HPV according to PCR results using the consensus primers. These samples contained cervical abnormalities enriched with inflammation (n = 107), cervical intraepithelial neoplasia (CIN) I (n = 19), CINII-III (n = 9) and cervical cancer (n = 31). Of the 166 HPV positive samples as determined by PCR analysis, 151 were further typed by PCR-SBT using 19 pairs of genotype-specific primers. Using this method, 17 different HR-HPV genotypes were identified. The most frequently observed HPV genotypes were HPV16 (44.0%, 73/166), 53 (28.9%, 48/166), 52 (25.3%, 42/166), 58 (22.3%, 37/166) and 35 (17.5%, 29/166). The proportions of single and multiple infections in the HPV-positive specimens were 34.9% and 65.1%, respectively. Multiple HPV types were most prevalent in the inflammatory state (63.0%), followed by cervical cancer (24.1%), CINI (11.1%), and CINII-III (1.9%). The results of our data analyses suggested that i) multiple HPV infection is not necessarily correlated with the severity of cervical abnormalities; and ii) among the multiple HPV infections, double infections combined with HPV16 is the most common. In addition, L1 full-length sequences of the top five high-risk HPV genotypes were amplified and sequenced. According to the L1 sequence of the epidemic genotypes that were amplified, we found that these genotypes contained the sequence point mutation, and that some of these genotypes further showed amino acid modifications. These results provide a basis for the construction of a polyvalent vaccine that is suitable for use in the XUAR, even in economically challenged communities located in China.

## Introduction

Cervical cancer is the second most common malignancy among women and the leading cause of cancer-related death among females worldwide [1, 2]. Epidemiological studies have shown that human papillomavirus (HPV) is the main cause of cervical cancer and precancerous lesions [3]. To date, over 100 HPV genotypes have been identified [4], and research studies have revealed that the pathogenicity of various strains of HPV is significantly different from each other [5]. Among the various types of HPV, the persistent infection of high-risk HPV (HR-HPV) is closely related to the occurrence of cervical intraepithelial neoplasia (CIN) and cervical cancer [6]. According to epidemiological and molecular biology reports, at least 19 HPV genotypes have been identified as HR-HPV (HPV16, 18, 26, 31, 33, 35, 39, 45, 51, 52, 53, 56, 58, 59, 66, 68, 70, 73 and 82) [7]. These genotypes mostly belong to oncogenic HPV, including alpha-9 (16, 31, 33, 35, 52 and 58) and alpha-7 (18, 39, 45, 59 and 68), which can cause HPV related genital tract tumors [8]. A woman can be repeatedly infected with HPV during her lifetime, which can also co-infect multiple HPV types [9]. In a pooled analysis of 15 areas worldwide, the prevalence of multiple HPV infection averaged about 25% and ranged from 18.5%–46% among HPV-positive women [10–12].

Prospective studies have shown that infection with multiple HR-HPV acted synergistically in cervical carcinogenesis [13]. Additionally, cancers with multiple HPV could be more resistant to therapy than those with a single infection. Munagala et al [14] reported that the treatment failure rate of cervical cancer patients with multiple HPV infections is five times that of patients with a single HPV infection. Therefore, the detection of HR-HPV infection, especially for major multiple HR-HPV infections, has become a key issue in the development of cervical lesion, and the epidemiological status of the population by HR-HPV genotyping method.

In China, approximately 135,000 new cases of cervical cancer are diagnosed each year and roughly 50,000 of these women die of cervical cancer annually [15]. The prevalence of cervical cancer has a clear increasing trend, with the incidence rate increasing by 2%–3% per year [16]. Xinjiang Uygur Autonomous Region (XUAR), Northwest China, has a relatively poor health condition that exists with the highest incidences of cervical cancer (490–527 out of 100,000 cases in southern XUAR) [17]. However, recent epidemiological data regarding cervical cancer, especially in northern region of XUAR, remains unclear. To elucidate these reports, the goals of this study were: i) to investigate the prevalence of HR-HPV in cervical abnormalities, and ii) to explore the correlation between multiple HPV infections of HR-HPV and cervical abnormalities; and iii) to analyze the nucleic acid variability of cervical cytology samples in order to evaluate the efficiency of vaccines that are currently available and designed to protect against HPV infections in XUAR.

## Methods

### Study Group

The research was conducted from January 2014 to December 2014. A total of 2,046 women were enrolled in the program entitled “The Xinjiang Production and Construction Corps Cervical Cancer Screening Study (XPCCCCSS).” The cytological examination of the collected Pap smears was carried out and defined according to the Bethesda classification [18]. Four hundred and twenty-eight women who presented abnormal cytological results were subjected to histological examination of their biopsies, which were conducted at two pathology laboratories. These biopsy samples were classified into 318 cases of inflammation, 42 cases of CINI, 9 cases of CINII-III, and 49 cases of cervical cancer. The mean age ± standard deviation and age range in each histological grade was as follows: inflammation, 39.76±9.7 years (18–72 years); CINI, 37.3±11.4 years (20–69 years); CINII-III, 48.4±6.8 years (31–69 years); and cervical cancer, 48.5±9.5 years (31–69 years). According to the exclusion criteria, women who were pregnant or less than 3 months post-partum, HIV-seropositive, had a history of hysterectomy, or were administered previous treatment for cervical cancer were ineligible for the study.

All eligible women who came to the screening had the details of the study explained to them, and written consent was obtained from all participants. The study was approved by the Gynecology and Pathology Departments of the First Affiliated Hospital, School of Medicine, Shihezi University.

### Sample Collection

From the cervix, cervical exfoliated cell specimens were collected from each participant using a Cyto-Brush (QIAGEN, Gaithersburg, MD, USA) for HPV DNA assays. According to the age of the participant and the stage of cervical cancer, the samples were divided into five groups (<25, 25–34, 35–44, 45–54, and ≥55).

### HPV DNA Detection

Four-hundred and twenty-eight abnormal cytological samples were stored in standard transport medium (STM), and tested by polymerase chain reaction–sequencing based typing (PCR-SBT). The consensus primer, MY09/MY11 [19], was used to amplify the fragments of capsid L1 gene of HPV. A total of 19 pairs of HR-HPV genotype-specific primers were respectively used to amplify each positive sample [7]. DNA from HeLa cells was used as the positive control and double distilled water (ddH_2_O) was used as the negative control. Beta-globin gene was used as the housekeeping gene to confirm the integrity of the DNA material extracted from the specimens. All primers were synthesized by Beijing Huada Inc., Beijing, China. DNA was extracted from the residual standard transport medium (STM)-stored cervical specimens using the TIANamp Genomic DNA kit (TianGen, Beijing, China) in accordance with the recommendations of the manufacturer. The reaction system was used as previously described [7, 19]. HPV genotypes were analyzed by sequencing (Beijing Huada Inc., Beijing, China). The resulting sequences from the HPV genotypes were compared with the reference sequences found in the centralized databases using BLAST (http://www.ncbi.nlm.nih.gov/BLAST/). If a sequence matched the reference sequence of a certain HPV subtype with ≥ 90% similarity, the sequence would be recognized to be the corresponding subtype.

### Amplification of L1 full-length sequences for prevalent HPV genotypes

The sequences of the most prevalent HPV genotypes were amplified as described in S1 File.

## Results

Among the 428 cervical samples analyzed in this study, 166 were found to be HPV-positive DNA as detected using consensus primer. In addition, we found 107 inflammation cases, 19 CINI cases, 9 CINII-III cases, and 31 cervical cancer cases. The prevalence and genotype distribution of the cervical abnormalities are shown in Table 1. Of 166 HPV-positive samples, 151 samples were successfully classified into 17 different HPV genotypes (HPV16, 18, 26, 31, 33, 35, 39, 45, 51, 52, 53, 56, 58, 59, 66, 68 and 73). The most common HPV genotypes were HPV16 (44.0%, 73/166), 53 (28.9%, 48/166), 52 (25.3%, 42/166), 58 (22.3%, 37/166) and 35 (17.5%, 29/166). The distribution and occurrence frequency of different HPV genotypes at different grades of abnormal cervical samples are listed in Table 1. Among them, HPV16, 53, 58 and 52 were the dominant types in the inflammation group, HPV16, 53, 52 and 58 were the dominant types in CINI, HPV33 and 53 were the dominant types in CINII–III. Meanwhile, in the cervical cancer group, the infection rate of HPV16, 52, 53 and 58 were highest. Among the 151 cases, the multiple HPV infections were observed in a total of 109 (65.7%) cases, while single HPV infection was found in 42 (25.3%) samples. Among the 108 cases of multiple HPV infections, the proportion gradually decreased with the increased number of infection genotypes, with 56 cases of double infection, 33 cases of triple infection, 15 cases of quadruple infection, 2 cases of pentagonal infection, and 2 cases of hexagonal infection.

**Table 1:** Distribution and occurrence frequency of different HPV genotypes at different grades of abnormal cervical samples.

	Inflammation(N = 318,74.3)	CINI(N = 42,9.8%)	CINII-III(N = 19,4.4%)	Cervical cancer(N = 49,11.4%)	Total(N = 428)
HPV (+)(n,%)	(107, 33.6)	(19, 45.2)	(9, 47.4)	(31, 63.3)	166
type					
16	45 (27.1)	10 (6.0)	1 (0.6)	17 (10.2)	73 (44.0)
18	5 (3.0)	0	0	2 (1.2)	7 (4.2)
26	1 (0.6)	0	0	3 (1.8)	4 (2.4)
31	7 (4.2)	2 (1.2)	1 (0.6)	0	10 (6.0)
33	12 (7.2)	1 (0.6)	3 (1.8)	0	16 (9.6)
35	18 (10.8)	2 (1.2)	1 (0.6)	8 (4.8)	29 (17.5)
39	9 (5.4)	1 (0.6)	0	4 (2.4)	14 (8.4)
45	3 (1.8)	1 (0.6)	1 (0.6)	0	5 (3.0)
51	5 (3.0)	0	0	1 (0.6)	6 (3.6)
52	25 (15.1)	6 (3.6)	1 (0.6)	10 (6.0)	42 (25.3)
53	28 (16.9)	8 (4.8)	2 (1.2)	10 (6.0)	48 (28.9)
56	11 (6.6)	0	0	4 (2.4)	15 (9.0)
58	26 (15.7)	3 (1.8)	0	8 (4.8)	37 (22.3)
59	4 (2.4)	1 (0.6)	1 (0.6)	0	6 (3.6)
66	8 (4.8)	0	0	2 (1.2)	10 (6.0)
68	3 (1.8)	0	0	2 (1.2)	5 (3.0)
73^X*^	1 (0.6)5 (3.0)	04 (2.4)	05 (3.0)	01 (0.6)	1 (0.6)15 (9.0)
Single HPV infection	26 (15.7)	7 (4.2)	3 (1.8)	5 (3.0)	41 (24.7)
Multiple HPV infection	68 (41.0)	12 (7.2)	2 (1.2)	26 (15.7)	108 (65.1)
Double infection	35 (21.1)	8 (4.8)	1 (0.6)	12 (7.2)	56 (33.7)
Triple infection	21 (12.7)	3 (1.8)	1 (0.6)	8 (4.8)	33 (19.9)
Quadruple infection	10 (6.0)	1 (0.6)	0	4 (2.4)	15 (9.0)
Quintet infection	2 (1.2)	0	0	0	2 (1.2)
Sextuple infection	0	0	0	2 (1.2)	2 (1.2)

Footnotes to Table 1: Abbreviations: CIN: Cervical intraepithelial neoplasia, HPV: human papillomavirus

X *: HPV type X denotes cervical samples that were positive according to the consensus primer system, but were not amplified by 19 pairs of genotype-specific primers.

Total surpass amount is 166, since women with multiple infections are counted at least twice.

**Fig 1:**
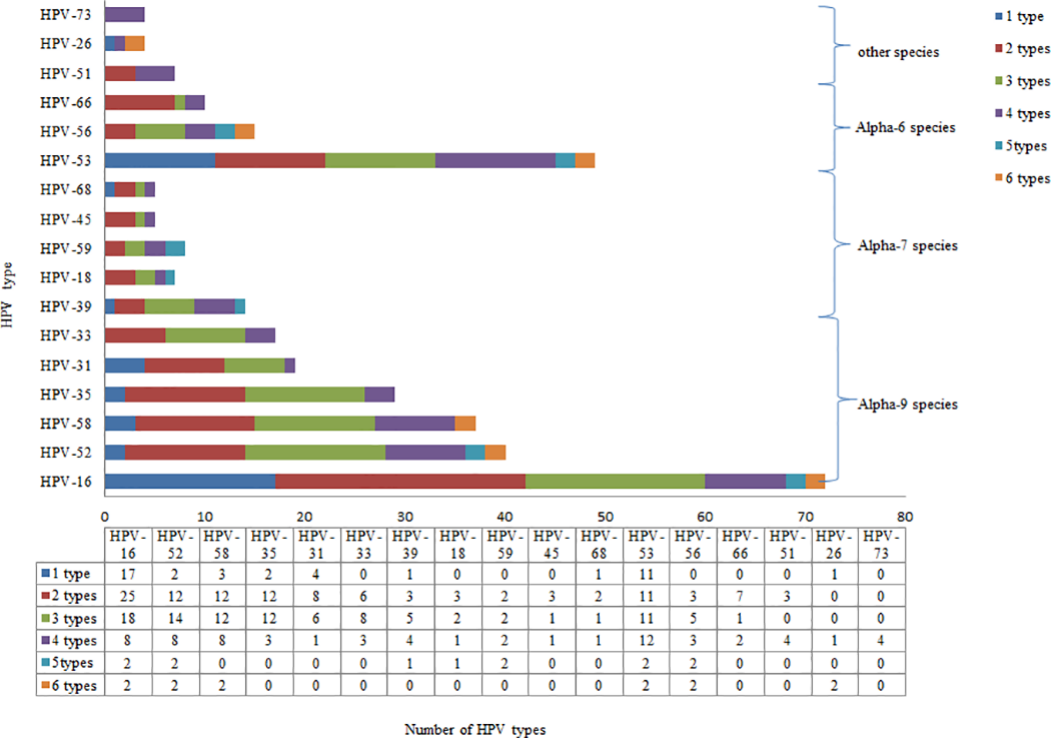
HPV type distribution among 166 HPV-positive cervical specimens.

Furthermore, in the 151 successfully genotyped samples, HPV16 combined infections were the most common types of infections, which were respectively involved in 44.6% (25/56), 54.6% (18/33), 53.3% (8/15), 100% (2/2) and 100% (2/2) of the double, triple, quadruple, pentagonal and hexagonal infections. HPV52 combined infections were the second most common type of combined infections (shown in Fig 1). The incident rates of HPV26 and 73 were relatively lower, which appeared in hexagonal infections and quadruple infections, respectively.

**Fig 2:**
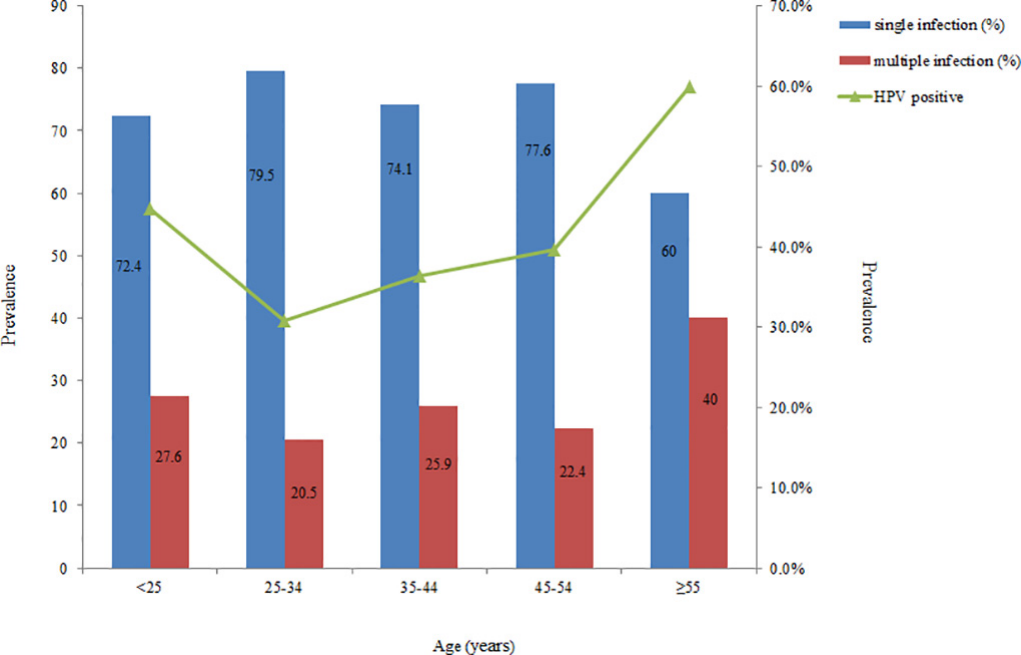
Correlation between multiple HPV infection rate and HPV positive rate according to the age of the patients.

The incidence of single HPV infection and multiple HPV infections, as well as the relationship between HPV-positive rate and age are shown in Fig 2. Among them, the infection rate of HPV was the highest in women at the age of 55 or older, followed by women who are younger than 25 years. Single HPV infection rate was the highest in the age group of women who were 25–34 years (79.5%). The rate of multiple HPV infections and the HPV-positive rate were the highest in the age group of women who were 55 years or older (40%).

The L1 full-length sequences of HPV16, 18, 52, 53, 58 that are found between the existing HPV vaccine strains and the prevalent HPV strains found in the United States of America were compared with the HPV genotypes obtained in this study. We observed that the HPV genotypes identified in this study contained sequence point mutations as indicated by BLAST, and that some of these mutations further led to amino acid modifications (S1 Table).

## Discussion

With the development of molecular epidemiology, biological risk factors for abnormal cervical incidence are mainly related to HPV infection. In this study, a total of 166 samples were found to be positive for HPV by PCR-SBT. HPV infection in samples having a background of inflammation, CINI, CINII–II, and cervical cancer were present in 33.6%, 45.2%, 47.4%, and 63.3% of these cases, respectively, which suggested that the positive rate of HPV increased with the severity of the pathological diagnostic grade. Such results indicate that HPV infection is closely related to the occurrence and development of CIN, which is consistent with the previous reports [20, 21]. The positivity rate of HPV increased with the severity of the pathological diagnostic grade, we found that the positive rate of HPV is 63.3% among cervical cancer cases using PCR. We believe that this occurrence is attributed to a theory known as “hit and run.” The previous studies indicated HPV might incite transformation in cells that subsequently lost their HPV DNA sequences during carcinogenesis [22–24]. In addition, the reason for the relatively low positive rate of HPV in cervical cancer cases may be due to the geographical environment and the selection criteria for the samples [25].

The distribution of HPV genotypes, especial HR-HPV genotypes, shows diversity in different countries. It is crucial to understand the distribution of HPV genotypes for designing prophylactic vaccines that are effective among geographically-specific populations in the future. In the present study, a total of 17 HR-HPV genotypes, namely HPV16, 18, 26, 31, 33, 35, 39, 45, 51, 52, 53, 56, 58, 59, 66, 68 and 73, were found. Of the 166 HPV-positive samples, HPV16 (44.0%, 73/166), 53 (28.9%, 48/166), 52 (25.3%, 42/166), 58 (22.3%, 37/166) and 35 (17.5%, 29/166) were the top five genotypes observed in this study. According to the grades of cervical abnormalities, HPV16, 53, 58 and 52 were the major HPV genotypes in inflammation, CINI and cervical cancer, while HPV33 and 53 were majorly involved in CINII–III cervical lesion.

Meta-analysis from the World Cancer Research Center indicated that HPV16, 31, 51, and 53 were the most prevalent HPV genotypes [26]. The data from South Korea showed that HPV16, 58, 18, and 52 were the most prevalent HPV genotypes [27]. Moreover, some reports demonstrate that HPV58, 52 and 53 were also the most common HPV genotypes [28, 29]. In the present study, the incidence of HPV18 infection was low, which may be attributable to the regional and racial differences or relatively poor sanitation conditions. Although the number of CINII–III samples was relatively small in the current study, we observed that infection by the HPV33 genotype was the highest. As HPV33 is a pathogenic HPV genotype leading to malignant cervical lesions, it is crucial to perform further investigations to elucidate our findings [30].

The popular opinion of many reports indicated that i) multiple HPV infections were associated with the grades of cervical abnormalities, and ii) the cervical cancer risk of patients who suffered from multiple HPV infections was higher than those with single HPV infection. However, there existed controversial conclusions in other reports. For example, Muñoz *et al*. [31] summarized 11 case control studies conducted between 1985 and 1997 in nine countries, and found that there was no significant difference in the risk of cervical cancer between multiple HPV infections and single HPV infection. In addition, a study regarding cervical cancer and CINII–III patients in South Africa revealed that multiple HPV infections were not correlated with the severity level of cervical cancer, and didn’t increase the incidence of cervical cancer [32]. In the present study, the positive rates of multiple HPV infections were highest in inflammatory cervical samples (41.0%, 68/166), followed by cervical cancer (15.7%, 26/166), CINI (7.2%, 12/166), and CINII–III (1.2%, 2/166). The incidence of multiple HPV infections indicated that multiple HPV infection was not related to the grades of cervical abnormalities.

Age is one of the most important risk factors for HPV infection [33]. Similar to a previous report [34], a U-shaped curve for HPV prevalence versus age was also observed in our study. The HR-HPV infection and the rate of multiple HPV infections in the group of women age 55 and older was the highest, followed by women younger than 25 years (shown in Fig 2). This result might be due to: i) more frequent sexual encounters among young women, which coexists with an immune system that has not been sensitized [35]; and ii) menopausal hormones have obvious fluctuations resulting from decline in ovarian function, which causes the physiological nature of the body decreased immune response with aging and the immune system to function in a disorderly manner, and have the diminished ability to eliminate and inhibit the virus, which further enhances susceptibility to form a persistent infection or to activate the virus during the incubation period, and even form a multiple infection [36, 37]. Therefore, our findings suggest that the Chinese government, especially in resource-poor communities, should pay more attention to the population age of women who are <25 and ≥55 years old for cervical cancer screening and HPV genotyping.

Currently, the application of L1 virus-like particle vaccines, including Gardasil®, Cervarix® [38], and 9-valent HPV6/11/16/18/31/33/45/52/58 vaccine after Phase II evaluation [39], is a promising measure against infection of HPV16, 52, 58 and 18. Here, we compared the L1 full-length sequence diversity between L1 sequences from vaccines and L1 sequences from HPV-positive samples from women in XUAR. All of the DNA sequences obtained from our study were deposited into GenBank (Accession No: KU721777-KU721779, KU721786-KU721793, and KU721799-KU721801). The results revealed that the L1 sequences of HPV16, 18 and 52, 53 and 58 contained obvious differences at the level of the amino acids. Variations in the amino acids may result from the difference in HPV type L1 immunogenicity. For future investigations, it is necessary to evaluate the overall efficacy of HPV vaccination before the introduction of existing HPV vaccines or novel HPV multivalent vaccines into high-prevalent region of China, especially in the XUAR.

Collectively, our findings demonstrated that: i) a total of 17 HR-HPV genotypes were found, and HPV16, 53, 52, 58 and 35 were the top five genotypes; ii) multiple HPV infections remained an important factor for cervical lesions, as evident in two age groups: women who were 55 years of age and older, and women who were younger than 25; and iii) L1 full-length sequences of HPV16, 18 and 52, 53 and 58 displayed obvious differences between the L1 sequences derived from the examined vaccines and from the HPV-positive samples from women in XUAR at the level of amino acids.

## Supporting Information

S1 File PCR protocol for the detection of capsid L1 gene of HPV16, 18, 52, 53, 58 of cervical cytology specimens in northern region of XUAR, northwest of China. (DOC)

S1 Table Amino acid change based on HPV L1 when being compared with the reference amino acid. (DOCX)
