# Supplementary material for: Prevalence of High-Risk Human Papillomavirus (HR-HPV) Genotypes and Multiple Infections in Cervical Abnormalities from Northern Xinjiang, China
Source: PLoS One. 2016 Aug 5;11(8):e0160698. doi: 10.1371/journal.pone.0160698 (PMC4975475; doi:10.1371/journal.pone.0160698)
Supplement: S1 File — (DOC) [file pone.0160698.s001.doc]

**S1 File**

**PCR protocol for the detection of the L1 major capsid protein gene of HPV16, 18, 52, 53, 58 of cervical cytology specimens, northern Xinjiang, China**

The PCR equipment was a TechneTC-412 thermal cycler, Barloworld Scientific, Cambridge, UK.

**1. PCR amplification of *HPV16, 58 L1* gene sequences from cervical cytology samples**

Each reaction consisted of 1 μL of HPV DNA (50 ng) and 12.5 μL of a PCR mix containing 50 mM KCl, 10 mM Tris-HCl (pH 8.3), 1.5 mM MgCl_2_, 250 μM of each dNTP, 40 pmol of each primer (*HPV16,58*), and 1.0 U of *Taq* DNA polymerase (TaKaRa Taq Version 2.0, Takara, Dalian, China).The cycling conditions consisted of an initial 5-min denaturation at 94°C, followed by 30 cycles at 94°C for 45s, 50°C for 45 s, and 72°C for 1 min, with a final extension at 72°C for 7 min.

**2. PCR amplification to detect the L1 major capsid protein gene of *HPV18* form the cervical cytology samples**

The PCR amplifications were performed in a 25-μL reaction volume. The reaction mixture contained 0.75 μmol/L of each primer (rrf-O), 250 μM of each dNTP, and 1.0 U of *Taq* polymerase (TaKaRa Taq Version 2.0, Takara, Dalian, China). The cycling conditions consisted of an initial 5-min denaturation at 95°C, followed by 35 cycles at 94°C for 45 s, 55°C for 45 s, and 72°C for 1min, with a final extension at 72°C for 8 min.

**3. PCR amplification to detect the L1 major capsid protein gene of *HPV52* from the capsid cytology samples**

The PCR amplifications were performed in a 25-μL reaction volume. The reaction mixture contained 0.75 μmol/L of each primer, 250 μM of each dNTP, and 1.0 U of *Taq* polymerase (TaKaRa Taq Version 2.0, Takara, Dalian, China). The cycling conditions consisted of an initial 5-min denaturation at 93°C, followed by 36 cycles at 93°C for 1min, {[ (52°C for 1min)-L1,L2]; [ (57°C for 1min)-L3,L4] and 72°C for 1 min, with a final extension at 72°C for 10 min.

**4. PCR amplification to detect the L1 major capsid protein gene of *HPV53* from the capsid cytology samples**

The PCR amplifications were performed in a 25-μL reaction volume. The reaction mixture contained 0.75 μmol/L of each primer, 250 μM of each dNTP, and 1.0 U of *Taq* polymerase (TaKaRa Taq Version 2.0, Takara, Dalian, China). The cycling conditions consisted of an initial 5-min denaturation at 95°C, followed by 38 cycles at 95°C for 45s, 57°C for 45s and 72°C for 2min, with a final extension at 72°C for 10 min.

**Nucleotide sequences of the primers used for the identification of the L**1major capsid protein gene

| Gene | Primer | Sequence (5’-3’) | Reference |
| --- | --- | --- | --- |
| HPV16 | HPV16-1F  HPV16-1R | ATAGTTCCAGGGTCTCCA  AGTCCATAGCACCAAAGC | 1 |
|  | HPV16-2F  HPV16-2R | GAACACTGGGGCAAAGGATC  TACAATGAATAACCACAACA |  |
| HPV18 | HPV18-1F  HPV18-1R | GTAACGGTCCCTTTAACCTCCTC  CATTGTCCCTAACGTCCTCAG | 2 |
|  | HPV18-2F  HPV18-2R | AAGTTCCCATGCCGCCACGTCTAAT  AGAGCCACTTGGAGAGGGAGAATAC |  |
|  | HPV18-3F  HPV18-3R | GCTCTATTGTTACCTCTGACTCC  ATTACTTCCTGGCACGTACACGCAC |  |
| HPV52 | HPV52-1F  HPV52-1R | CCATTACCTTCGTTACCCACA  AGGATGCCCACTAATACCC | 3 |
|  | HPV52-2F  HPV52-2R | GCTTGGAAATCGGTAGGG  GCAGTATTGCCAGAGTTAGACC |  |
|  | HPV52-3F  HPV52-3R | AGGGTCTAACTCTGGCAATAC  CCTGTAGCCCTGCCTGTA |  |
|  | HPV52-4F  HPV52-4R | ATGAAAATTTTAAGGAATACC  ACATACATAACATGCAAACAAC |  |
| HPV53 | HPV53-F  HPV53-R | GGGACATCCTTATTACCCCATTT  AGTAGAAGCAGAGCGTTTTTTAG | This study |
| HPV58 | HPV58-1F  HPV58-1R | GTGTCATTGGAACCTGGTCCA  GCCAAGTTTTCCAGCCCTATT | 1 |
|  | HPV58-2F  HPV58-2R | GCCAGTGAACCTTATGGGGAT  TTTGCGTTTGGTGGATGGT |  |

References

1. Yue YF, Yang HY, WU K, Yang LJ, Chen JY, et al. Genetic Variability in L1 and L2 Genes of HPV-16 and HPV-58 in Southwest China. PLoS One. 2013; 8 (1): e55204.doi: 10.1371/journal.pone 0055204. PMID: 23372836; PubMed Central PMCID: 3555822

2. Shen MJ, Ding XP, Li TJ, Chen GY, Zhou X. Sequence variation analysis of HPV-18 isolates in southwest China. PLoS One. 2013; 8 (2): e56614.  doi: 10.1371/journal.pone.0056614 PMID: 23451059; PubMed Central PMCID: 3581518

3. Luo ZY, Chen Q, Yang LY, Lin M, Chen MR. Analysis of L1 gene polymorphism of human papillomavirus type 52 from Chaozhou area of China. Chin J Lab Diagn. 2013; 17: 793-798.
