# Supplementary material for: Prevalence of High-Risk Human Papillomavirus (HR-HPV) Genotypes and Multiple Infections in Cervical Abnormalities from Northern Xinjiang, China
Source: PLoS One. 2016 Aug 5;11(8):e0160698. doi: 10.1371/journal.pone.0160698 (PMC4975475; doi:10.1371/journal.pone.0160698)
Supplement: S1 Table — (DOCX) [file pone.0160698.s002.docx]

**Supporting Table 1:** Amino acid change based on HPV L1 when being compared with the reference amino acid

| HPV genotype | Accession NO | Reference Accession NO  (originated country) | Mutant position (amino acid) |
| --- | --- | --- | --- |
| HPV16 | KU721786 | ST-HPV16 | 228(D-H)  292(A-T)  449(S-.^*^)  466(^*^.-D) |
|  | KU721787 |  | 228(D-H)  292(A-T)  449(S-.^*^)  466(.-D) |
|  | KU721788 |  | 228(D-H)  292(A-T)  449(S-.^*^)  466(^*^.-D) |
| HPV18 | KU721789 | ST-HPV18 | 25(R-Q)  64(L-M)  91(P-R)  149(T-N)  344(P-R)  399(P-R)  535(G-R) |
|  | KU721790 |  | 25(R-Q)  91(P-R)  344(P-R)  399(P-R)  539(K-T) |
|  | KU721791 |  | 25(R-Q)  91(P-R)  344(P-R)  399(P-R) |
| HPV52 | KU721792 | HQ537740(USA) | 386(N-K) |
|  | KU721793 |  | 386(N-K) |
| HPV53 | KU721799 | EF546482(USA) | 58(T-A) |
|  | KU721800 |  | 145(I-V)  262(S-A) |
|  | KU721801 |  | 58(T-A)  145(I-V)  262(S-A) |
| HPV58 | KU721777 | HQ5377762(USA) | 10(V-A)  150(L-F)  159(G-S)  163(T-P)  292(T-K)  296(P-A)  299(N-D)  311(G-V)  325(I-M)  378(D-G)  383(N-D) |
|  | KU721778 |  | 5(L-F)  10(V-A)  144(I-V)  150(L-F)  159(G-S)  163(T-P)  292(T-K)  296(P-A)  299(N-D)  311(G-V)  325(I-M)  378(D-G)  383(N-D)  410(V-I)  420(N-D)  422(D-N) |
|  | KU721779 |  | 10(V-A)  144(I-V)  159(G-S)  163(T-P)  259(D-G)  292(T-K)  296(P-A)  299(N-D)  311(G-V)  376(K-T)  378(D-G)  383(N-D)  410(V-I)  420(N-D)  422(D-N) |

.* deletion in this position in the level of amino acid.
